# Layered Al/CuO Thin Films for Tunable Ignition and Actuations

**DOI:** 10.3390/nano10102009

**Published:** 2020-10-12

**Authors:** Ludovic Salvagnac, Sandrine Assie-Souleille, Carole Rossi

**Affiliations:** CNRS, LAAS, University of Toulouse, 7 Avenue du Colonel Roche, F-31400 Toulouse, France; salvagnac@laas.fr (L.S.); souleille@laas.fr (S.A.-S.)

**Keywords:** nanothermite, pyroMEMS, nanoenergetics, reactive thin film

## Abstract

Sputter-deposited Al/CuO multilayers are used to manufacture tunable igniters and actuators, with applications in various fields such as defense, space and infrastructure safety. This paper describes the technology of deposition and the characteristics of Al/CuO multilayers, followed by some examples of the applications of these energetic layers.

## 1. Introduction

Energetic materials are widely used to suddenly generate high amounts of thermal or mechanical energy under an electrical, mechanical or thermal stimulus. As typical energetic materials, nanothermites, which contain Al and oxide, have attracted much attention as they exhibit not only better combustion efficiencies and better ignitability compared to traditional explosives, but also their reaction outputs can be tuned, thanks to the selections of fuels, oxidizers, architectures and reactant size, allowing multiple actions. Among nanothermites, sputter-deposited Al/CuO multilayers represent interesting energetic thin film nanomaterials for tunable ignition, to replace old hot-wire ignitors.

In that context, our research group demonstrated several miniature pyrotechnical ignition devices integrating Al/CuO multilayers within microelectromechanical systems (MEMS) based microheaters, called pyroMEMS, for civilian and military applications such as triggering the inflation of airbags, micro-propulsion systems, and arm and fire devices.

This paper briefly reviews the technological process, focusing on the sputter-deposition technique, presents the properties of Al/CuO multilayered films, details the fabrication process flow of a micro-igniter, and present examples of applications of pyroMEMS.

## 2. Al/CuO Multilayered Nanothermites

Two decades ago, the introduction of nanotechnologies enabled the emergence of a new class of energetic materials called nanothermites that could play a great role in future society. Nanothermites use raw materials that can be found in abundance, are low-cost and non-polluting (green materials). They also exhibit better combustion efficiencies and better ignitability compared to typical CHNO energetic mixtures, while being safer. Importantly, the reactions and ability to trigger specific actions can be tailored by varying the size and composition of the oxide, allowing multiple applications. Focusing on thermite multilayers, the overwhelming majority of works concern Al/CuO systems, as they feature an exceptionally high-energy release with gaseous production.

### 2.1. Multilayers Deposition

**Figure 1:**
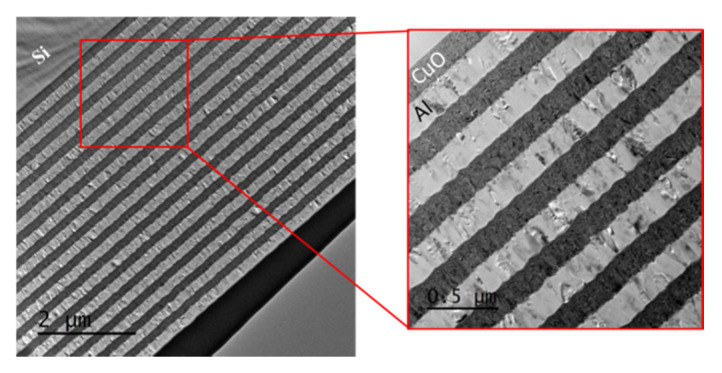
Cross-sectional transmission electron micrographs of the Al/CuO multilayer obtained by magnetron sputtering.

Al/CuO thermite multilayers are mainly produced by the sputter-deposition technique [1,2,3,4,5], as this provides excellent control over the layering and stoichiometry. Cupric oxide thin film is synthesized using the direct current (DC) reactive magnetron sputtering technique in an oxygen-enriched environment. To produce Al/CuO multilayered films, layers of Al and CuO are deposited on top of each other in an alternating fashion (see Figure 1) without venting the chamber. However, after each layer of deposition, the sample stage is cooled at ambient temperature for 600 s. At each interface between the cupric oxide and Al layers, an intermixing layer of 4–8 nm is present that forms during the deposition itself.

Depositions parameters have been published several times and can be found in [6,7].

### 2.2. Ignition and Reaction Properties 

Applying an external source of energy locally on the multilayer results in an increase in the temperature of the multilayer section directly in contact with the heated surface. This can be done by electrostatic discharge [8], mechanical impact [9], laser irradiation [10], electrical heating (spark) [11] or thermal hot points [12], the latter of which is the widest used. After being ignited, the multilayers react by a self-sustained propagating reaction following the chemistry of Equation (1).

2Al + 3CuO → Al_2_O_3_ + 3Cu (+ ∆H) (1)

In other words, after the temperature is raised locally and rapidly to a characteristic onset reaction temperature, the reaction enthalpy (∆H) spreads into neighboring unreacted portions of the film, leading to a self-sustained reaction wave, which is highly luminous. 

As the exothermic chemical reactions are controlled by the outward migration of oxygen atoms from the CuO matrix towards the aluminum layers, the reaction rate strongly depends on the reactant spacing (bilayer thickness). The sustained combustion rate is commonly characterized with a standard high-speed camera under ambient pressure [5,7].

**Figure 2:**
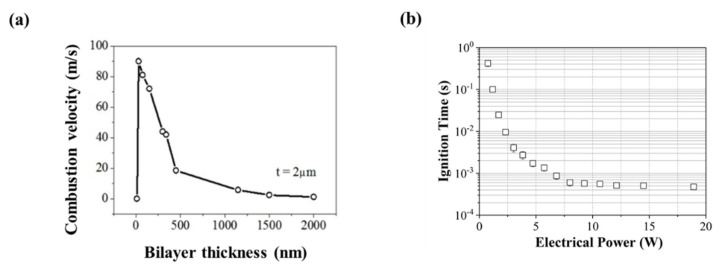
(**a**) Sustained combustion velocity in air of free-standing multilayers as a function of bilayer thickness (total thickness t = 2 µm); (**b**) ignition time as a function of the electrical power sent through the micro-heater for 5 bilayers of Al/CuO.

The combustion velocity increases for thinner bilayers, with a maximum at ~90 m/s for free-standing foils (see Figure 2a). However, the reaction stops suddenly for extremely small bilayer thicknesses, which is due to the presence of interfacial layers, as described earlier, which reduces the amount of stored energy, i.e., reaction enthalpy, ∆H.

## 3. PyroMEMS Integration and Characterization 

**Figure 3:**
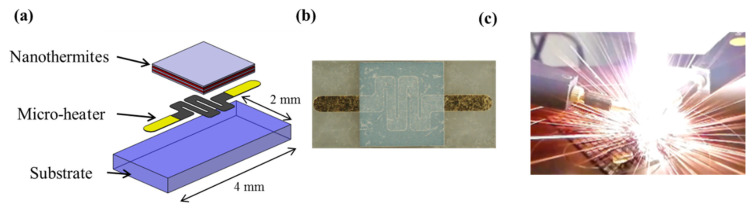
(**a**) 3D schematic representation of a pyroMEMS; (**b**) photo of the pyroMEMS; (**c**) photo during the nanothermite reaction.

When integrated onto a device/substrate, the widest and simplest ignition method for igniting an Al/CuO multilayered thin film is using hot-wire, which functions via local metallic thin film resistance. A pyroMEMS is mainly composed of a substrate, thin metallic resistance and the Al/CuO nanothermites (see Figure 3a).

To minimize the ignition energy, the substrate must be a good thermal insulator. We chose 500 µm thick 4-inches glass substrates AF32 (Schott AG, Mainz, Germany). After being cleaned in oxygen plasma to remove surface contaminants, 300 nm thick titanium followed by 300 nm thick gold layers are evaporated onto the glass substrate, and the filament is patterned using the lift-off technique. Then the gold is chemically etched and patterned to define the Ti resistance and Au electrical pads. Finally, Al/CuO multilayers are sputter-deposited through a silicon shadow mask. A photo of a pyroMEMS thus manufactured is presented in Figure 3b.

We characterized that, for any heating surface areas (micro-heater sizes), the ignition time (delay between the time of application of the electrical power and the emission of the light) rapidly decreases when the ignition power density increases, until an asymptotic value, defining the minimum response ignition time, which is a characteristic of the multilayered film itself. 

As an illustration, Figure 2b provides the ignition time versus the ignition power for a thermite made of 15 300 nm thick 1:1 Al/CuO bilayers. Below a certain electrical power, no ignition occurs, regardless of the duration of application of the power. This is simply explained by the fact that, below this ignition power threshold, the electrical energy supplied to the micro-heater is not sufficient to compensate for the energy lost by conduction through the substrate, by radiation from the exposed surface, or by convection in the air. The multilayer cannot reach its ignition temperature, also known as the onset reaction temperature. 

This threshold highly depends on the heating surface area, and detailed results can be found in [12].

## 4. Examples of Applications

To date, igniters are the widest investigated applications and are widely used to trigger the inflation of airbags, micro-propulsion systems, and the arm and fire devices used in missiles, rockets and any other ordnance systems. Traditionally, igniter technology consists of a metallic hot-wire (Ni/Cr, et al.) or bridge wire in contact with a secondary or primary explosive. The fabrication and integration of explosives in igniters requires extreme precaution due to their high sensitivity to electrostatic discharge, friction or shock.

Al/CuO multilayers can efficiently replace the hot-wire and primary explosive. Other advantages of replacing traditional hot-wire with pyroMEMS are as follows: (1) The overall integration is easier, requiring only the electrical connections of the pyroMEMS, whereas traditional pyrotechnical igniters require at least two steps (hot-wire and primary pyrotechnic composition deposition). (2) The Al/CuO multilayers are safe and less sensitive to the environment. (3) Ignition threshold and reaction output (flame temperature and gaseous products) can be easily tuned by changing the Al/CuO bilayer thickness and stoichiometry (bilayer thickness ratio).

**Figure 4:**
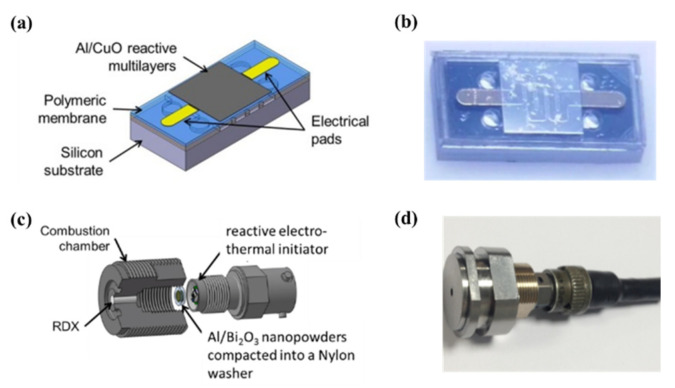
(**a**) Schematic and (**b**) photo of the reactive electro-thermal initiator on Epoxy/PET membrane. Chip dimension is 4 mm × 2 mm. (**c**) Schematic and (**d**) photo of one miniature detonator integrating Al/CuO multilayers as the initiator.

Taton et al. [13] first reported the design, realization and characterization of hot-wire ignition integrating Al/CuO multilayers (see Figure 4) for use in pyrotechnical systems for space applications. The reactive Al/CuO multilayered thin film resides on a 100 μm thick epoxy/polyethyleneterephtalate (PET) membrane to insulate the reactive layer from the bulk substrate. When current is supplied, Al/CuO reacts and the products of the reaction produce sparks that can ignite any secondary energetic composition, such as RDX (hexogen). The authors demonstrated a 100% success of ignition over a 0.25–4 A firing current range, corresponding to 80–244 μJ, and with response times ranging from 2 to 260 μs. Then, Glavier et al. [14] used this igniter to cut and propel a thin metallic foil and ignite RDX in a detonation. Authors showed that a stainless-steel flyer of 40 mg can be properly cut and propelled at velocities calculated from 665 to 1083 m/s, as a function of the RDX’s extent of compaction and ignition charge. The impact of the flyer can directly initiate the detonation of an RDX explosive, which is very promising in terms of removing primary explosives from detonators. A schematic view and photo of one miniature detonator is given in Figure 4.

**Figure 5:**
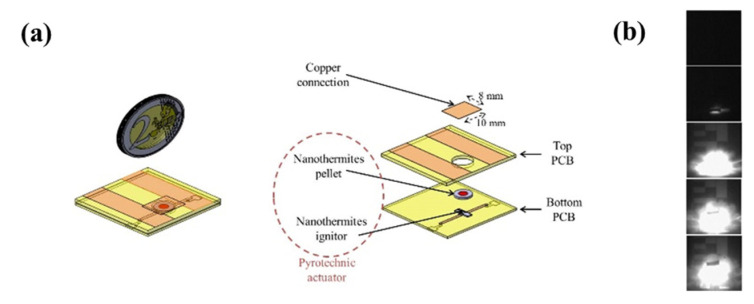
(**a**) 3D schematic of the nanothermite-based circuit breaker with exploded views and (**b**) snapshots of high speed images taken for the tested circuit breaker. The time between each picture is 100 µs. Taken from [15].

More recently, Nicollet et al. [12] developed the Al/CuO multilayer igniter concept for several substrates, and simplified the fabrication process to adapt it to airbag initiation. Additionally, our team has demonstrated miniature one-shot circuit breakers [15], based on the combustion of a nanothermite. Each device is simply made from two assembled printed board circuits (PCBs) to define a hermetic cavity in which an Al/CuO multilayer initiator chip ignites—in less than 100 µs—a few milligrams of nanothermite, to cut a thick copper connection (see Figure 5). The authors demonstrated the good operation (100% success rate) with a response time of 0.57 ms, which is much lower than the response time of classical mechanical circuit breakers (>ms).

## 5. Conclusions

Sputter-deposited Al/CuO multilayers, composed of alternating Al and CuO nanolayers with total thicknesses ranging from 1 to tens of µm, present an opportunity for tunable ignition and actuation. The interest in and integration of igniter applications have been demonstrated for different applications, such as in inflators, aerospace or actuators.
